# Indocyanine Green Fluorescence Navigation in Pediatric Hepatobiliary Surgery: Systematic Review

**DOI:** 10.3390/children12070950

**Published:** 2025-07-18

**Authors:** Carlos Delgado-Miguel, Javier Arredondo-Montero, Julio César Moreno-Alfonso, Isabella Garavis Montagut, Marta Rodríguez, Inmaculada Ruiz Jiménez, Noela Carrera, Pablo Aguado Roncero, Ennio Fuentes, Ricardo Díez, Francisco Hernández-Oliveros

**Affiliations:** 1 Pediatric Surgery Department, Fundación Jiménez Díaz University Hospital, 28040 Madrid, Spain; marta.rodriguezruiz@estudiante.uam.es (M.R.);; 2 Institute for Health Research IdiPAZ, La Paz University Hospital, 28046 Madrid, Spain; francisco.hernandezo@quironsalud.es; 3 Pediatric Surgery Department, Complejo Asistencial Universitario de León, 24008 Castilla y León, Spain; jarredondo@saludcastillayleon.es; 4 Pediatric Surgery Department, Navarra University Hospital, 31008 Pamplona, Spain; moreno.151076@e.unavarra.es; 5 Research Hotbed in General Surgery and Subspecialties, Universidad El Bosque, Ak. 9 #131a-2, Bogotá 110311, Colombia; igaravis@unbosque.edu.co; 6 Pediatric Surgery Department, La Paz University Hospital, 28046 Madrid, Spain

**Keywords:** liver, bile ducts, indocyanine green, near-infrared fluorescence, children, pediatric

## Abstract

**Introduction**: Near-infrared fluorescence (NIRF) imaging with indocyanine green (ICG) is now widely regarded as a valuable aid in decision-making for complex hepatobiliary procedures, with increasing support from recent studies. **Methods**: We performed a systematic review following PRISMA guidelines, utilizing PubMed, CINAHL, and EMBASE databases to locate studies on the perioperative use ICG in pediatric hepatobiliary surgeries. Two independent reviewers assessed all articles for eligibility based on predefined inclusion criteria. We collected data on study design, patient demographics, surgical indications, ICG dosing, timing of ICG injection, and perioperative outcomes. **Results**: Forty-three articles, including 930 pediatric patients, from 1989 to 2025 met the inclusion criteria for narrative synthesis in our systematic review, of which 22/43 (51.2%) were retrospective studies, 15/43 were case reports (34.9%), 3/43 (7.0%) were experimental studies, and the other three were prospective comparative studies (7.0%). The current clinical applications of ICG in hepatobiliary pediatric surgery include bile duct surgery (cholecystectomy, choledochal cyst, biliary atresia), reported in 17 articles (39.5%), liver tumor resection, reported in 15 articles (34.9%), liver transplantation, reported in 6 articles (14.6%), and liver function determination, reported in 5 articles (12.2%). **Conclusions**: ICG fluorescence navigation in pediatric hepatobiliary surgery is a highly promising and safe technology that allows for the intraoperative localization of anatomic biliary structures, aids in the identification and resection of liver tumors, and can accurately determine hepatic function. The lack of comparative and prospective studies, and the variability of the dose and timing of administration are the main limitations.

## 1. Introduction

The application of near-infrared fluorescence (NIRF) imaging with indocyanine green (ICG) has become increasingly popular in pediatric surgery and is now regarded as a valuable tool for decision-making during complex procedures, supported by growing evidence in the literature [1,2]. ICG has several advantages that have contributed to the exponential increase in its use in different areas of pediatric surgery over the last decade. ICG is a non-ionizing, as it does not use radiation, making it safe for pediatric populations; it has a short half-life, being rapidly eliminated from the body, which allows for multiple applications within a single procedure; and it offers high specificity, with fluorescence that is easily distinguishable in imaging systems, even in deep tissues [3,4]. This fluorescence occurs when ICG, a water-soluble dye, is activated by light of a specific wavelength within the NIRF spectrum (~820 nm) and visualized using specialized optics and camera systems [5].

In recent years, some narrative and systematic reviews have been published on the use of ICG in different fields of application within pediatric surgery, such as urology, oncology, and gastrointestinal or colorectal surgery [6,7]. The usefulness of ICG in hepatobiliary surgery has also been reported, as it is almost exclusively metabolized by the liver and excreted in bile, with different applications, doses, and administration times, although, to date, the existing literature has not been analyzed in this regard. During this type of surgery, the real-time visualization of ICG fluorescence overlay is highly beneficial for comprehending vascular or biliary anatomy and conducting dissections safely, not only in patients with unusual anatomy but also in those with significant visceral fat, which can complicate the identification of anatomical structures [8]. These developments underscore the need to standardize protocols for ICG use specific to hepatobiliary surgical procedures, including its indications, dosing, timing, and methods of administration. This systematic review outlines the benefits, current uses, and potential future developments of NIRF imaging with ICG in pediatric hepatobiliary surgery.

## 2. Methods

### 2.1. Search Strategy

A systematic review was conducted in accordance with the Preferred Reporting Items for Systematic Reviews and Meta-Analyses (PRISMA) guidelines [9,10]. The research question was structured using the Population, Intervention, Comparator, Outcome (PICO) framework: ‘What are the clinical applications of ICG in pediatric hepatobiliary surgery?’ A comprehensive literature search was performed across three electronic databases (PubMed, CINAHL, and EMBASE) in December 2024, with an updated search conducted on 1 May 2025 by two authors (CDM and IGM). To identify relevant studies, the PubMed search employed the following MeSH terms: [“indocyanine green” OR “fluorescence-guided”] AND [“children” OR “pediatric”]. Similar Emtree terms were applied for the CINAHL and EMBASE database searches to ensure consistency. The primary outcome was to identify the main indications for the use of ICG in pediatric hepatobiliary surgery. Secondary outcomes were to analyze the dose used, route of administration, timing of use, and safety of the drug (associated adverse effects). The protocol of this systematic review is registered in the PROSPERO database (registration CRD420251057059).

### 2.2. Eligibility Criteria

Studies were considered eligible for inclusion if they involved patients under 18 years of age and addressed the perioperative use of ICG in hepatobiliary surgery. To qualify, publications needed to be full-text articles presenting original clinical data related to at least one of the following pediatric hepatobiliary procedures: cholecystectomy, biliary atresia, choledochal cyst, liver tumors (hepatoblastoma or hepatocellular carcinoma), liver function assessment, or liver transplantation. Only studies conducted in human subjects and written in English were included.

Exclusion criteria comprised editorials, review articles lacking original clinical data, commentaries, conference abstracts, studies involving animals or exclusively adult populations, and those unrelated to gastrointestinal surgery. Articles that included both pediatric and adult patients were retained only if the mean or median patient age was below 18 years, or if sufficient data on the pediatric subgroup permitted independent analysis. Duplicate entries were removed, and remaining records were screened initially by title and abstract, removing those in which it was not specified that ICG was used in hepatobiliary surgery in pediatric patients. Full-text versions of potentially relevant studies were subsequently reviewed in detail to confirm compliance with inclusion criteria. Review articles were temporarily retained to allow for a manual screening of their reference lists, ensuring comprehensive literature coverage through a backward snowballing approach. This strategy enhanced the identification of all pertinent studies aligned with the predefined eligibility criteria.

### 2.3. Data Collection and Synthesis

Information on study characteristics, patient demographics, sample size, age at the time of surgery, underlying conditions, surgical techniques employed, ICG dosing, timing of ICG injection, postoperative complications, and clinical outcomes was extracted from each included publication. Data collection was independently carried out by two reviewers (CDM and IGM) using a standardized Microsoft Excel™ (2007, Redmond, WA, USA) spreadsheet. The entire review process—comprising screening, eligibility assessment, and data extraction—was independently performed by both investigators. Additionally, relevant references cited within the selected studies were manually screened to identify further eligible articles. Any disagreements were resolved by discussion until a consensus was reached. Due to substantial heterogeneity across studies and the limited sample size, a meta-analysis was not feasible. Instead, a descriptive synthesis is provided, summarizing the current pediatric hepatobiliary surgical indications for ICG use, as well as reported timing and dosing practices. Ethical approval was not required given the nature of this systematic review.

### 2.4. Quality Appraisal

To evaluate the methodological rigor of the included studies, the Methodological Index for Non-Randomized Studies (MINORS) was employed [11]. This tool comprises 12 distinct items, each representing a specific quality criterion tailored for comparative research. Each item is rated as 0 (not mentioned), 1 (mentioned but insufficient), or 2 (mentioned and sufficient), with a maximum possible score of 24. One reviewer initially assessed the studies using this index, and a second reviewer verified the evaluation. In cases of disagreement, a third reviewer was consulted to reach a consensus. Studies were classified as high quality if they achieved a score of 17 or above, while those scoring below 17 were categorized as low quality.

## 3. Results

**Figure 1:**
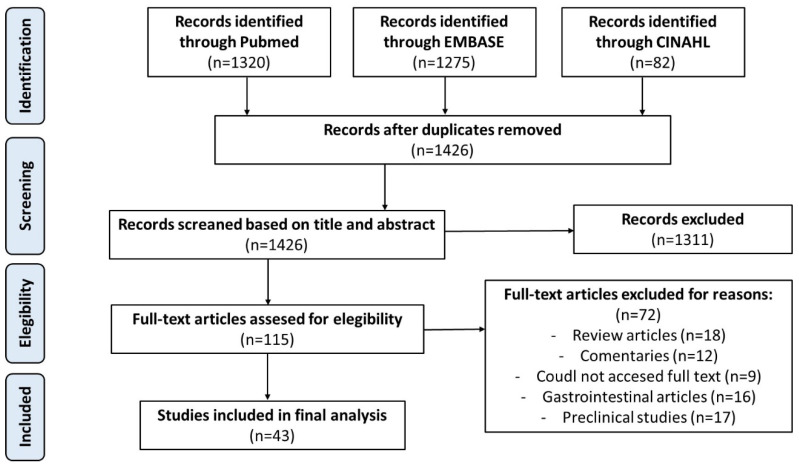
PRISMA flowchart.

An initial total of 1426 records were identified through database searches, from which 43 studies ultimately fulfilled the inclusion criteria and were incorporated into the narrative synthesis of this systematic review. A cumulative cohort of 930 pediatric patients treated between 1989 and 2025 was analyzed. Among the included studies, 22 (51.2%) were retrospective analyses, 15 (34.9%) were case reports, 3 (7.0%) were observational experimental investigations, and the other 3 were prospective comparative studies (7.0%). The reported clinical applications of ICG in pediatric hepatobiliary surgery were distributed as follows: bile duct procedures—including cholecystectomy, choledochal cyst excision, and biliary atresia—featured in 17 studies (39.5%), which are summarized in Table 1; liver tumor resections were described in 14 articles (34.9%); liver transplantation was addressed in 6 studies (14.0%); and assessments of liver function were discussed in 5 studies (11.6%). Quality analysis according to the MINORS scale revealed only two articles scoring 20 points (high quality), while the rest were between 12 and 14 points (low quality); these results are shown in detail in the Supplementary Material (Excel File). The process of article selection, along with the reasons for exclusion, is illustrated in the PRISMA flow diagram in Figure 1.

### 3.1. Cholecystectomy

The use of ICG in pediatric laparoscopic cholecystectomy was first introduced by Graves et al. in 2017, who described the direct intracholecystic injection of ICG using a catheter-assisted technique [12]. This approach, resembling conventional cholangiography but using ICG as a contrast agent diluted with aspirated bile to a low concentration (0.025 mg/mL), enabled clear visualization of the biliary anatomy in 11 patients without any adverse events. Subsequently, the intravenous administration of ICG became more widely adopted, either intraoperatively or several hours before surgery [13,17]. Calabro et al. evaluated ICG fluorescence cholangiography in 31 pediatric patients, successfully identifying both the common bile duct and hepatic duct. They reported a significantly shorter operative time compared to conventional contrast cholangiography (105 vs. 164 min), suggesting potential procedural efficiency [15]. Esposito et al. have been publishing their accumulated experience over the last few years in different articles, and recently compared the use of ICG in 90 patients versus 83 cases with conventional laparoscopic cholecystectomy without ICG, in which they found 7.5% of anatomical anomalies, both vascular and biliary [16]. Importantly, the rate of intraoperative complications was significantly lower in the ICG group (0% vs. 12%, *p* < 0.005), with complications in the control group including one major bile duct injury requiring reoperation (Clavien-Dindo IIIb), gallbladder injuries, and bleeding from Calot’s triangle or the hepatic bed. Additionally, ICG imaging has been tested in complex anatomical cases such as gallbladder duplication, as reported by Bryant et al. [14]. In one such case, the presence of an accessory right hepatic duct arising from the cystic duct required a conventional cholangiogram, as ICG fluorescence alone was insufficient to fully delineate the biliary structures.

### 3.2. Biliary Atresia (BA)

There are two main uses of ICG in BA: it serves both as a diagnostic and prognostic tool. Its use was first introduced intraoperatively during Kasai portoenterostomy procedures by Japanese surgical teams. In 2015, Hirayama et al. reported its intraoperative application to help identify the porta hepatis, noting that the liver exhibited diffuse fluorescence uptake, while no extrahepatic structures showed any fluorescence [18]. They classified three distinct fluorescence patterns at the porta hepatis: spotty fluorescence, diffuse weak fluorescence, and diffuse strong fluorescence.

In 2019, Yanagi et al. published their experience using real-time near-infrared fluorescence cholangiography with ICG to evaluate biliary flow during surgery for biliary atresia. They applied this technique in 10 patients and detected fluorescence in the hilar microbile ducts and exudate, which helped determine the optimal level for dissection of the fibrous tissue at the liver hilum [19]. Moreover, certain studies have analyzed stool fluorescence both before and after surgery, suggesting that variations in fluorescence intensity in fecal samples may serve as an indicator of changes in bile excretion. Zhao et al. recently investigated the diagnostic and prognostic potential of ICG quantification in nine neonates with BA, assessing fluorescence intensity in the liver and hepatic hilum both before and after dissection of fibrotic tissue, as well as in the jejunum following portoenterostomy [21]. To evaluate short-term outcomes, they tracked ICG clearance in the stool and used jaundice resolution (total bilirubin < 2 mg/dL) at 1–3 months postoperatively as a clinical marker. In four patients, ICG was fully cleared from the body within approximately two weeks, with no fluorescence detected in the stool thereafter. The remaining five patients took longer than two weeks. This allowed the authors to estimate bile drainage efficacy based on how long fluorescence persisted in the stool after surgery, concluding that variations in intrahepatic ICG signal and postoperative clearance time might be associated with clinical prognosis. Lim et al. employed preoperative ICG administration by intravenously injecting it into neonates with jaundice and subsequently quantifying ICG fluorescence in soiled diapers over the following 24 h. They demonstrated that patients in whom no fluorescence was detected in the diapers were later confirmed to have biliary atresia during exploratory laparoscopy [24].

ICG has also increasingly been used to evaluate the extrahepatic bile ducts during exploratory laparoscopy in neonates with cholestasis. All these authors agree that, intraoperatively, none of the patients with biliary atresia exhibited fluorescence within the gallbladder [17,23,25]. The largest reported series is that of Shirota et al., who studied 57 patients suspected of having BA and compared bile visualization using ICG with conventional cholangiography [20]. In 43 cases with BA, fluorescence was observed only in the liver, with no fluorescence detected in the gallbladder or common bile duct, showing a 100% sensitivity when compared to cholangiography, while in the remaining 14 patients, the sensitivity of ICG fluorescence for diagnosing BA exclusion was 64.3%. TKJ et al. described the usefulness of ICG-guided fluorescence for directing the removal of fibrous tissue at the porta hepatis. As the dissection advances, the observed fluorescence becomes more intense, helping to indicate the appropriate depth for excision [17]. Wang et al. used ICG in a case of type II cystic biliary atresia, administering it one hour prior to surgery. This enabled dissection distal to the cyst, revealing an atretic common hepatic duct that exhibited faint fluorescence under near-infrared imaging. Following the hepatic portoenterostomy, bile flow from the liver to the intestine through the anastomosis was clearly visualized in fluorescence mode [22].

### 3.3. Choledochal Cyst (CDC)

The application of ICG in CDC surgery is the most recent to emerge, with only four publications available since 2022, three of which are case reports. Masuya et al. were the first to report its usefulness in a case involving congenital biliary dilation classified as type IVa according to the Todani system, combined with a rare hepatic artery branching pattern, where the posterior superior pancreaticoduodenal artery ran along the right side of the dilated common bile duct and drained directly into the posterior segment of the right hepatic lobe [8]. Intraoperative ICG fluorescence enabled complete laparoscopic artery dissection without injury, allowing for safe cyst excision and hepaticojejunostomy without complications. Delgado-Miguel et al. later documented the benefit of ICG in visualizing the intrahepatic extent of a type Ic CDC in an 18-month-old girl, facilitating accurate delineation of the biliary anatomy before duct transection, which is particularly valuable in fusiform cysts, where complete removal is essential but carries a risk of damaging accessory hepatic ducts [27]. Onishi et al. demonstrated the use of ICG injected into the gallbladder under laparoscopic guidance in a 9-year-old girl with pancreaticobiliary maljunction confirmed by intraoperative cholangiography [26]. Fluorescence imaging enabled clear identification of the distal end of a type Ia choledochal cyst embedded within the pancreatic tissue, helping to avoid injury to that region, which otherwise would have appeared as ICG leakage. More recently, TK et al. presented the first series involving six pediatric patients with CDC (four type IVa, one type IVb, and one type Ic), all of whom underwent laparoscopic cyst excision followed by hepaticoduodenostomy [17]. In this series, ICG was administered intravenously 12 to 18 h prior to surgery. The authors noted fluorescence interference from bowel loops in two cases and reported one instance where the leakage of fluorescent fluid from the duodenum facilitated the prompt detection of an iatrogenic injury.

### 3.4. Liver Tumors

The use of ICG in hepatic tumors was first reported in 2014 by Mitani et al., who described its application in the intraoperative identification of a liver nodule located on the hepatic surface 11 months after resection of a hepatoblastoma [28]. This was followed in 2015 by Yamamichi et al., who reported its use in two cases of hepatoblastoma—one primary and one recurrent—located between the previous surgical resection margin and the diaphragm. In both cases, ICG fluorescence enabled complete tumor removal with clear surgical margins [29].

Subsequently, Yamada et al. (2019) reported the use of fluorescence-guided navigation in 13 hepatectomies, where ICG enabled both the identification of the primary tumor and the detection of nodules at the resection margin, with no false negatives observed [30]. Additionally, ICG has proven valuable in identifying additional lesions that were not detected on preoperative imaging, as described by Shen et al. [31]. In a separate study, Shen et al. conducted a retrospective analysis of 23 pediatric hepatoblastoma resections, in which intravenous ICG (0.5 mg/kg) was administered 48 to 72 h before surgery. Fluorescence imaging effectively outlined the tumor margins, enabling complete resections with negative margins in all cases. Postoperatively, the surgical specimens were examined under fluorescence, revealing three distinct uptake patterns: total, partial, and ring fluorescence [32].

Souzaki et al. highlighted the usefulness of ICG in identifying residual tumor at the resection margin and detecting diaphragmatic invasion during open surgery in four patients with primary hepatoblastoma [33]. Qui et al. described, in 2022, the first ICG-guided laparoscopic resection of hepatoblastoma in seven patients, administering intravenous ICG at a dose of 0.5 mg/kg 48 h prior to surgery. The fluorescence of the liver tissue was utilized to inform the resection approach, and no tumor recurrence was observed during a median follow-up of 24 months [34]. Liu et al. applied ICG fluorescence imaging in 22 patients with hepatoblastoma, observing hyperfluorescence in all cases, which included both total and partial fluorescence patterns. ICG-guided navigation facilitated minimally invasive procedures in three patients—one via laparoscopy and two with robotic assistance. They reported that 17% of the cases showed suspicious lesions at the resection margin or in other hepatic lobes, which were histologically confirmed as tumors. Notably, no ICG-negative tumors were identified, resulting in a false-negative rate of 0% [35]. However, they also reported a high false-positive rate (83%) in suspicious lesions that were analyzed and ultimately showed no tumor cells. In most cases, they associated this with the administration of ICG 48 h before the intervention.

Cho et al. reported that ICG fluorescence imaging in children with hepatoblastoma was both feasible and safe, aiding in precise tumor demarcation and improving the accuracy of complete tumor resection. A median safety margin of 6 mm (range, 0–11 mm) was achieved, with no residual lesions detected in the liver on follow-up computed tomography [36]. These findings are consistent with those reported by Feng et al. in eight cases of hepatoblastoma, where after resection of the tumor and all ICG-positive lesions, no fluorescence was observed in the remaining liver tissue. Pathological analysis confirmed complete tumor removal with negative surgical margins in all cases [37]. Lake et al. reported a case in which no hepatic tumor uptake of ICG was observed in a patient scheduled for a left hemihepatectomy, corresponding to a false-negative rate of 11.1% [38].

Finally, the intraoperative utility of ICG has also been reported in other types of hepatic tumors. Chung et al. utilized ICG fluorescence during the laparoscopic resection of a hepatocellular carcinoma in a 9-year-old girl, where the tumor margins became more clearly defined under fluorescence imaging, as the lesion appeared significantly brighter than the surrounding tissue [39]. Whitlock et al. described its use in one case of hepatocellular carcinoma and another case of malignant rhabdoid tumor [40], and Shen et al. used it to intraoperatively identify an inflammatory myofibroblastic liver tumor. It has also been used in liver tumors that do not uptake ICG [41]. Yamamoto et al. used ICG to differentiate an undifferentiated embryonal sarcoma of the liver, which did not fluoresce, from the remaining healthy liver parenchyma, which did fluoresce [42]. Table 2 provides a summary of studies that have explored the use of ICG for detecting hepatic tumors intraoperatively.

### 3.5. Liver Transplant

ICG fluorescence navigation has been used in living donor liver transplantation to better identify the donor ductal anatomy in laparoscopic donor hepatectomy, as reported by several authors [43,44,45]. Intravenous injection allows for prompt visualization of the left hepatic artery and biliary duct in less than 10 s, followed by immediate visualization of the sheet where the biliary ducts are located in the hilar plate, confirming an important anatomical landmark. Li et al. compared ICG fluorescence in 21 patients with cholangiography via the bile duct stump of segment IV (11 cases) in whom a left lateral sectionectomy in living donor liver transplantation was performed, with no differences between the two groups [46]. In addition, other uses of ICG in liver transplantation surgery have been reported. Kisaoglu et al. used ICG fluorescence to detect ischemic areas in the left lobe on postoperative day 4 of a deceased donor liver transplantation. After ICG intravenous injection, no fluorescence was observed in the necrotic area during reperfusion of the transplanted graft, and a thrombosis of the left hepatic artery was detected [47]. Lemoine et al. described the usefulness of ICG fluorescence for the intraoperative detection of a small non-anastomotic cut on day 9 after split liver transplantation, located in the surface bile leaks, which was not identified with 4.5× loupe magnification [48]. Table 3 lists the main uses of ICG in liver transplantation.

### 3.6. Liver Function

The first studies on the hepatic metabolism of ICG were published more than 30 years ago. Evans et al., in 1988, compared the clearance of ICG among different age groups, showing that it was higher in younger children versus adolescents and adults [49]. Subsequently Kubota et al. reported the correlation between ICG clearance and prognosis in patients with biliary atresia after Kasai hepaticoportojejunostomy. In those patients with ICG clearance figures higher than 0.19, a lower development of hepatic complications such as gastrointestinal bleeding or esophageal varices was observed [50]. The Spanish group led by Quintero et al. investigated the prognostic value of the indocyanine green plasma disappearance rate (ICG-PDR) in pediatric patients with acute liver failure. Their findings demonstrated its potential as a reliable indicator for disease progression, enabling clinicians to stratify patients based on severity [51]. Nielsen et al. assessed a non-invasive method for measuring ICG clearance using pulse spectrophotometry and compared it with conventional spectrophotometric analysis based on serial blood sampling, revealing a strong linear relationship between the two approaches [52]. More recently, Ficerai-Garland et al. assessed the association between ICG clearance and liver function in 124 children with liver disease, of whom 40% had undergone a transplant. They observed linear regression of ICG clearance with other liver function parameters such as international normalized ratio (INR) or prothrombin-proconvertin clotting time [53]. An overview of ICG-based methods for liver function assessment is presented in Table 3.

## 4. Discussion

This systematic review compiles existing studies on the application of ICG fluorescence imaging in pediatric hepatobiliary surgery—an area that remains relatively unexplored in the literature to date. Previous reviews have predominantly addressed ICG usage in fields such as pediatric urology, oncologic surgery in children, and gastrointestinal interventions. Nonetheless, the growing range of hepatobiliary indications in recent years underscores the necessity of an updated synthesis focused specifically on this surgical domain. Notably, 29 out of the 43 articles analyzed in this review were published within the past five years, highlighting the rapid and expanding adoption of ICG fluorescence in pediatric hepatobiliary procedures.

Regarding its surgical applications, ICG-guided fluorescence during cholecystectomy enables precise visualization of the extrahepatic biliary structures, particularly the junction between the cystic duct and the common bile duct. It also enhances the delineation of the dissection plane between the gallbladder and the liver. In the largest published cohort to date, successful visualization of the entire biliary tree was achieved in 98.8% of cases [16]. However, ICG-fluorescent cholangiography has limited utility in detecting common bile duct stones, and for this reason, conventional contrast-enhanced X-ray cholangiography remains the preferred diagnostic method in scenarios where biliary obstruction is suspected [14]. ICG has also demonstrated to be a safe tool in laparoscopic procedures for biliary conditions such as biliary atresia and choledochal cysts. It offers a radiation-free alternative to conventional intraoperative cholangiography by enabling the real-time visualization of the biliary anatomy and bile flow. This fluorescence-guided technique functions as a dynamic navigation aid during surgery. In addition, the overlay mode allows for the fusion of ICG fluorescence with a standard white-light image, enhancing anatomical orientation during dissection. Better visualization of the bile duct in patients with anatomical variants or abundant visceral fat may avoid negative consequences in these patients. Recent studies have indicated that the incidence of common bile duct (CBD) injuries during hepatobiliary surgeries is slightly higher in children (0.80%) than in adults (0.37%). This difference is likely attributed to the lower volume of pediatric cases and the comparatively limited experience of pediatric surgeons in managing hepatobiliary conditions [54]. In CDC surgery, the criteria for determining the transection plane remain unstandardized, particularly in cases without bile duct dilation and in patients with pancreaticobiliary maljunction, due to the challenges in precisely identifying this junction in small children. ICG fluorescence imaging facilitates clear delineation of both the intrahepatic (proximal) and intrapancreatic (distal) extents of the CDC, thereby reducing the risk of injury at these critical sites. Any such damage could be detected intraoperatively by the macroscopic observation of fluorescent fluid leakage. Furthermore, similar to its application in biliary atresia, ICG allows for the early detection of bile leaks at the biliary anastomosis site during hepaticojejunostomy.

While ICG-guided surgery offers high sensitivity for the detection of hepatoblastoma, enhancing the surgeon’s ability to accurately assess the tumor’s location and margins, a notable limitation is its tendency toward false-positive findings. Non-malignant hepatic conditions that have been reported to exhibit ICG fluorescence include regenerative nodules, bile duct hyperplasia, dysplastic lesions, chronic inflammatory changes, fibrotic areas, normal liver tissue, biliary plugs, and hepatic cysts [55]. When ICG is administered intravenously the day prior to surgery, its clearance from non-tumorous liver tissue may be insufficient, leading to the appearance of numerous false-positive nodules. This effect becomes more pronounced in patients with impaired liver function [29]. An additional technical constraint of ICG fluorescence imaging is its limited tissue penetration depth, which is approximately 5–10 mm. As a result, lesions situated deeper within the liver may go undetected with current imaging systems. To overcome this limitation, photoacoustic imaging is under investigation as a complementary modality, with the potential to visualize ICG accumulation while integrating data from intraoperative ultrasound [56].

In the context of liver transplantation, ICG fluorescence imaging proves valuable during both graft procurement and graft implantation procedures. During living donor hepatectomy, it aids in the accurate identification and delineation of vascular and biliary structures, minimizing the risk of injury during dissection. Additionally, it facilitates the detection of bile leaks on the liver surface following transplantation—an aspect that may be more difficult to assess than anastomotic leaks—and can also reveal vascular complications such as arterial or venous thrombosis during implantation or in the postoperative period. The usefulness of ICG in the analysis of liver function has been increasing over recent decades. From early studies reporting liver clearance in children and adults, we have moved on to correlating the prognosis of chronic liver diseases such as biliary atresia and even predicting the severity of acute liver failure. In addition, the validation of minimally invasive techniques such as pulse spectrophotometry has allowed us to extend the use of ICG as a parameter to indicate liver function similarly to laboratory parameters obtained by blood tests, such as INR or prothrombin–proconvertin clotting time.

Differentiating target anatomical structures—such as extrahepatic bile ducts or hepatic nodules—from the background fluorescence emitted by normal liver tissue is a frequent challenge in ICG fluorescence-guided surgery. Optimizing fluorescence quality depends on the careful adjustment of critical parameters including ICG dosage, the timing of administration, and surgical technique. The dosage of ICG used in pediatric hepatobiliary procedures typically ranges from 0.025 to 0.6 mg/kg, a margin well below the reported lethal threshold of 80 mg/kg, indicating a strong safety profile [26]. The most common route of administration is intravenous; however, it can also be directly injected into the gallbladder, mimicking traditional contrast-based cholangiography to enhance biliary tract visualization. Phenobarbital treatment may impair both the intensity and timing of ICG fluorescence, representing a relevant limitation that should be considered during surgical planning [57]. Evidence suggests that low ICG concentrations are sufficient to produce effective fluorescence imaging without compromising liver health, whereas elevated doses of ICG may impact hepatic function in pediatric patients [58]. The likelihood of adverse reactions following ICG administration is extremely low, with most incidents reported at doses exceeding 0.5 mg/kg, corresponding to an estimated incidence of just 0.003% [59,60]. As ICG contains a small quantity of iodine to facilitate its solubility in water, it is important to assess patients for a history of iodine hypersensitivity prior to administration. In individuals with known iodine sensitivity, premedication may be advisable. Nonetheless, reports have documented the safe use of ICG in patients with confirmed iodine allergies [61]. All reviewed studies consistently reported ICG administration to be well-tolerated, with no documented adverse reactions or clinically relevant complications.

In pediatric hepatobiliary surgery, the timing of ICG administration varies considerably, showing notable heterogeneity not only across different clinical indications but also among authors addressing the same condition. Following intravenous injection, ICG rapidly binds to plasma proteins and remains largely confined to the vascular system. It is then almost entirely taken up by hepatocytes and cleared into the bile at a rate of approximately 18–24% per minute. When administered intraoperatively, biliary excretion of ICG can be observed within minutes; however, hepatic parenchyma also exhibits fluorescence. Around 6–8 h after administration, the liver fluorescence begins to fade, while biliary tract fluorescence persists, allowing for clearer visualization of the bile ducts. This explains why, in the vast majority of hepatobiliary indications, it is usually administered preoperatively, in contrast to the use in gastrointestinal surgery, where it is mainly administered intraoperatively [4]. In cholecystectomy, ICG can be injected either intraoperatively or a few hours prior to the procedure, as rapid biliary excretion allows for timely visualization of the bile ducts. In contrast, procedures such as biliary atresia or choledochal cyst surgery require administration 12–24 h before the operation to reduce background fluorescence from the liver and intestinal loops, which can obscure biliary anatomy. A similar rationale applies to hepatic tumor resections, where most authors advocate for preoperative administration at least one day in advance to ensure optimal contrast between tumor and surrounding tissue. This variation is largely due to ICG’s pharmacokinetics: after hepatic uptake and biliary excretion, residual fluorescence in bowel loops can persist and contribute to non-specific signals. Delaying surgery to 48–72 h after injection allows this intestinal fluorescence to dissipate, improving the accuracy of intraoperative imaging [62].

A key limitation of this review lies in the methodological rigor of the included studies, which impacts the strength of the conclusions. This limitation is largely attributed to the nature of the included studies, most of which were either retrospective case series or individual case reports. The overall quality of evidence was affected primarily by the lack of non-exposed control groups, as only a few studies directly compared outcomes between patients who received ICG and those who did not. Common methodological issues inherent to such study designs—including limited generalizability, retrospective data collection, and susceptibility to publication bias—represent major barriers to broader clinical implementation of ICG in pediatric hepatobiliary surgery. Additionally, a meta-analysis could not be conducted due to the considerable heterogeneity among the included studies.

## 5. Conclusions

This systematic review is the first to comprehensively synthesize the available literature on the application of ICG fluorescence imaging in pediatric hepatobiliary surgery. The technique has demonstrated promising safety and utility, particularly in enhancing the intraoperative visualization of anatomical landmarks, which facilitates more precise dissection and reduces the risk of iatrogenic injury. Its clinical use spans a wide range of indications, including cholecystectomy, biliary atresia surgery, choledochal cyst excision, hepatic tumor resections, liver transplantation, and hepatic function assessment. Notably, ICG offers a radiation-free alternative to conventional intraoperative cholangiography, representing a significant advantage in pediatric populations. Furthermore, its potential to allow for the early detection of intraoperative bile leaks offers a valuable adjunct to surgical safety, especially in complex biliary reconstructions. However, certain limitations must be acknowledged. ICG fluorescence may yield false-positive signals, particularly in the presence of inflammation or background autofluorescence, and its capacity to detect deeply located lesions is inherently restricted due to the limited tissue penetration of near-infrared light. Additionally, the current evidence indicates that ICG may have limited sensitivity in identifying common bile duct stones. The primary limitation of the current body of evidence remains the scarcity of high-quality, prospective comparative studies. Such investigations are urgently needed to validate preliminary findings, establish standardized protocols regarding dosing and timing, and better delineate the precise indications and limitations of ICG use in pediatric hepatobiliary surgery.

## Figures and Tables

**Table 1:** Current clinical applications of ICG in hepatobiliary pediatric surgery.

References (Country, Year)	Indication for ICG NIRF Surgery	Study Design	No. Patients	Age (Range)	Timing of ICG Injection	ICG Dose (Route)
**Cholecystectomy**						
Graves et al. (USA, 2017) [12]	Illumination of relevant biliary system during laparoscopic cholecystectomy	Case series	11	Mean: 16 years	Intraoperative	0.025 mg/mL ICG-bile solution. (Injected inside the gall bladder)
Fernández-Bautista et al. (Spain, 2019) [13]	Identification of the bile duct during laparoscopic cholecystectomy	Case report	1	13 years	Intraoperative	0.2 mg/kg
Bryant et al. (USA, 2020) [14]	Identification of anatomic variant of duplicated gall bladder with an accessory right hepatic duct branching off the cystic duct	Case report	1	17 years	Intraoperative	Non-specified
Calabro et al. (USA, 2020) [15]	Identification of biliary tree anatomy during laparoscopic cholecystectomy	Case series	31	Mean: 15 years old (range 6–18 years)	After anesthesia induction	1 mL of a 25 mg/mL solution (IV)
Esposito et al. (Italy, 2024) [16]	Compare outcomes of laparoscopic cholecystectomy with or without ICG	Retrospective study (comparative)	173	Median: 12 years (4–17 years)	Median time: 15 h before surgery (range 8–20 h)	0.35 mg/kg (IV)
T K et al. (India, 2025) [17]	Visualization of the biliary anatomy	Prospective study	2	13 years both	2–6 h before surgery	0.5 mg/kg (IV)
***Biliary atresia (BA)***						
Hirayama et al. (Japan, 2015) [18]	Visualize the biliary flow during and after anastomosis	Retrospective study	5	Median: 42 days. (31–75 days)	24 h before surgery	0.1 mg/kg (IV)
Yanagi et al. (Japan, 2019) [19]	Observing the hilar microbile ducts during dissection	Retrospective study	10	Mean: 75 days (48–122 days)	24 h before surgery	0.5 mg/kg (IV)
Shirota et al. (Japan, 2022) [20]	Explore the use of intravenous ICG cholangiography for the exclusion of BA	Retrospective study	57	Median: 61 days (34–201 days)	Three times: 24 h before surgery, 1 h before surgery or both	0.05 mg/kg (IV)
Zhao et al. (China, 2022) [21]	Anatomical degree of hepatic portal fibrous tissue evaluation and prognostic correlation after surgery	Observational study	9	Mean: 73 days (32–112 days).	4–17 h before surgery	0.05 mg/kg (IV)
Wang et al. (China, 2023) [22]	Monitor bile flow during Kasai procedure (laparoscopic)	Case report	1	25 days	After induction of general anesthesia	0.05 mg/kg (IV)
Zhang et al. (China, 2024) [23]	Evaluation of the extrahepatic bile duct during exploratory laparoscopy	Retrospective study	16	Mean: 55 days (42–93 days)	12 h before surgery	0.3 mg/kg (IV)
Lim et al. (USA, 2024) [24]	Evaluation of ICG fluorescence in soiled diapers in neonates with jaundice during the 24 h following intravenous ICG injection	Prospective cohort study	22	Mean: 8 days	24 h before surgery	0.1 mg/kg (IV)
T K et al. (India, 2025) [17]	Visualize the biliary flow during and after anastomosis	Prospective study	9	Median 71 days (52–163 days)	18–24 h before surgery	0.5 mg/kg (IV)
Jaseel et al. (India, 2025) [25]	Identification of bile flow during Kasai procedure (open)	Retrospective study	11	Mean: 70 days (44–111 days)	12 h before surgery	0.1 mg/kg (IV)
**Choledochal cyst**						
Masuya et al. (Japan, 2022) [8]	Locate and preserve a variant of the right hepatic artery (congenital biliary dilatation type-IVa)	Case report	1	9 years	Intraoperative, before dissection of the dilated CBD	0.6 mg/kg (IV)
Onishi et al. (Japan, 2022) [26]	Detection of pancreaticobiliary maljunction with co-injection of bile and ICG (choledochal cyst type Ia)	Case report	1	4 years	Intraoperative, before dissection of the dilated CBD	Mix of 30% ICG and 70% bile (inside the gallbladder through catheter injection)
Delgado-Miguel et al. (Spain, 2023) [27]	Identification of the choledocal cyst margins (choledochal cyst type Ic)	Case report	1	18 months	8 h before surgery	0.5 mg/kg (IV)
T K et al. (India, 2025) [17]	Visualize the biliary anatomy	Prospective study	6	Median: 6 years (3–16 years)	12–18 h before	0.5 mg/kg (IV)

ICG, indocyanine green; NIRF, near-infrared fluorescence; IV, intravenous.

**Table 2:** Current clinical applications of ICG in liver tumors in children.

References (Country, Year)	Indication for ICG NIRF Surgery	Study Design	No. Patients	Age (Range)	Timing of ICG Injection	ICG Dose (Route)
**Hepatoblastoma**						
Mitani et al. (Japan, 2014) [28]	Identification of tumor recurrence in liver surface	Case report	1	32 months	48 h before surgery	0.5 mg/kg (IV)
Yamamichi et al. (Japan, 2015) [29]	Surgical navigation with ICG to improve complete resection	Case series	3	Mean: 3 years (1–6 years)	3–4 days before surgery	0.5 mg/kg (IV)
Souzaki et al. (Japan, 2019) [33]	Detection residual tumor and invasion of the diaphragm	Retrospective study	4	Median: 28 months (12–35 months)	60–138 h prior surgery	0.5 mg/kg (IV)
Yamada et al. (Japan, 2019) [30]	Fluorescence guided tumor resection	Retrospective study	12	Median: 12 months (8–168 months)	72 h before surgery	No data available about doses they used
Lake et al. (USA, 2021) [38]	Lesion identification and complete tumor resection	Retrospective study	9	Median: 3 years (1–12 years)	1–6 days before surgery	0.5 mg/kg (IV)
Cho et al. (South Korea, 2021) [36]	Demarcation of liver primary tumor	Retrospective study	17	Median: 18 months (4–140 months)	24–48 h before surgery	0.3 mg/kg (IV)
Shen et al. (China, 2022) [31]	Identification of margins for tumor resection	Retrospective study	16	Median: 15 months (8–134 months)	48–72 h before surgery	0.5 mg/kg (IV)
Whitlock et al. (USA, 2022) [40]	Intraoperative guidance for resection of primary pediatric liver tumors	Retrospective study	12	Median: 91 months (10–228 months)	24–96 h prior surgery	0.2–0.75 mg/kg IV
Feng et al. (China, 2022) [37]	Comparison of tumor fluorescence with false-positive nodules	Retrospective study	8	Median: no data (8–154 months)	1 day before surgery	0.1–0.2 mg/kg (IV)
Qiu et al. (China, 2022) [34]	Fluorescence-guided laparoscopic tumor resection	Retrospective study	7	Median: no data (13 days–36 months)	48 h before surgery	0.5 mg/kg (IV)
Shen et al. (China, 2023) [32]	Tumor resection Postoperative evaluation of the fluorescence	Retrospective study	23	Median: 26 months (5–80 months)	24–48 h before surgery	0.1 mg/kg (IV)
Liu et al. (China, 2023) [35]	Real-time fluorescence navigation during tumor resection	Retrospective study	22	Median: 32 months (15–45 months)	10 cases: 24 h before surgery 9 cases: 48 h before surgery 3 cases: 72 h before surgery	10 cases: 0.1 mg/kg (IV) 9 cases: 0.3 mg/kg (IV) 3 cases: 0.5 mg/kg (IV)
**Other liver tumors**						
Chung et al. (Hong Kong, 2020) [39]	Intraoperative tumor navigation for laparoscopic hepatectomy (hepatocellular carcinoma)	Case report	1	9 years	24 h before surgery	0.5 mg/kg (IV)
Yamamoto et al. (Japan, 2022) [42]	Identification of unaffected margins (undifferentiated embryonal sarcoma of the liver)	Case report	1	7 years	4 days before surgery	0.5 mg/kg (IV)
Whitlock et al. (USA, 2022) [40]	Intraoperative guidance for resection (hepatocellular carcinoma)	Retrospective study	1	8 months	24 h before surgery	0.5 mg/kg (IV)
Whitlock et al. (USA, 2022) [40]	Intraoperative guidance for resection (malignant rhabdoid tumor)	Retrospective study	1	5 years	24 h before surgery	0.2 mg/kg (IV)
Shen et al. (China, 2024) [41]	Intraoperative tumor identification (inflammatory myofibroblastic tumor)	Case report	1	55 months	No data	Non specified

ICG, indocyanine green; NIRF, near-infrared fluorescence; IV, intravenous.

**Table 3:** Update clinical applications of ICG in liver transplantation and in the study of liver function.

References (Country, Year)	Indication for ICG NIRF Surgery	Study Design	No. Patients	Age (Range)	Timing of ICG Injection	ICG Dose (Route)
**Liver transplant**						
Troisi et al. (Saudi Arabia, 2014) [43]	Optimize bile duct division in laparoscopic in left lateral sectionectomy	Case report	1	2 years	Intraoperative	0.1 mg/kg (IV)
Hong et al. (South Korea, 2018) [44]	Intraoperative visualization of bile duct anatomy for identification of the optimal division point	Case report	1	43 years	Intraoperative	Non-specified
Kisaoglu et al. (Turkey, 2020) [47]	Identifying alterations in graft perfusion	Case report	1	4 years	Intraoperative	0.05 mg/kg (IV)
Umemura et al. (Japan, 2021) [45]	Confirm the cutting line while performing laparoscopic left lateral sectionectomy	Retrospective study	5	Non specified	Intraoperative	Non-specified
Li et al. (China, 2023) [46]	Visualization of bile duct division in laparoscopic left lateral sectionectomy	Retrospective study (comparative)	21	Mean: 33 years	Intraoperative	2.5 mg/body (IV)
Lemoine et al. (USA, 2023) [48]	Localization of bile leak after liver transplantation	Case report	1	5 years	Intraoperative	0.5 mg/kg (IV)
**Liver function**						
Evans et al. (USA, 1989) [49]	Evaluate hepatic drug clearance in children and adults	Observational study	115	Mean age: 6.7 years, (0.9–17.8 years)	9–14 h before measurements	0.5 mg/kg (IV)
Kubota et al. (Japan, 1993) [50]	Evaluating hepatic function in postoperative biliary atresia patients	Retrospective study	19	Mean age: 4.9 years (2.3–8.3 years)	Every year after the surgery	1 mg/kg (IV)
Quintero et al. (Spain, 2014) [51]	Evaluate ICG plasma disappearance rate as a predictor of pediatric acute liver failure evolution	Prospective study	48	Survivors: 6.5 years (5–9 years) Non-survivors: 3 years (1.5–7)	Non specified	0.25 mg/kg (IV)
Nielsen et al. (Denmark, 2019) [52]	Compare ICG elimination kinetics measured by spectrophotometry of serial blood samples and by pulse spectrophotometry.	Observational study	87	Mean age: 11.9 years (0.6–19 years)	0, 5, 10, 15, and 20 min after measurements	0.25 mg/kg (IV)
Ficerai-Garland et al. (USA, 2025) [53]	Measure the ICG clearance obtained by pulse spectrophotometry	Observational study	124	Mean age: 11 years (6–15 years)	5, 10, 15 and 20 min after injection of ICG	0.25 mg/kg (IV)

ICG, indocyanine green; NIRF, near-infrared fluorescence; IV, intravenous.

## Data Availability

Code and other materials: Data collection form templates; data extracted from included studies; and any other materials used in the review can be requested from the corresponding author of this study.

## Supplementary Materials

The following supporting information can be downloaded at: https://www.mdpi.com/article/10.3390/children12070950/s1, MINORS_scores (version 1).
